# Association between kidney function and mortality among incident metformin users: a retrospective cohort study

**DOI:** 10.1186/1753-6561-6-S4-O46

**Published:** 2012-07-09

**Authors:** M Schorr, L Bresee, A Soo, B Hemmelgarn

**Affiliations:** 1 Department of Medicine, Royal College of Surgeons in Ireland; 2 Department of Community Health Sciences, University of Calgary, Canada; 3 Department of Medicine, University of Calgary, Canada

## Introduction

Metformin is first-line therapy for people with type 2 diabetes. Due to potential lactic acidosis it is contraindicated in patients with estimated glomerular filtration rate (eGFR) < 60 mL/min/1.73m2, although little is known about its use among patients with chronic kidney disease (CKD). The objective of this study was to determine the association between kidney function and mortality among incident metformin users.

## Methods

Databases of the Alberta Kidney Disease Network (http://www.akdn.info; Alberta, Canada) were used to identify a cohort aged 66 and older with diabetes who were incident metformin users from November 1, 2002 until March 31, 2008. The cohort was stratified on frequency of creatinine measurements prior to initiating metformin: none; a pre measurement only (6 months prior to metformin initiation); a post measurement only (within 1 year after); and both pre and post measurements. Patients were categorized by stage of CKD: 1 & 2 ( ≥ 60 mL/min/1.73m2; reference); 3a (45 - 59 mL/min/1.73m2); 3b (30 -44 mL/min/1.73m2); 4 & 5 (< 30 mL/min/1.73m2); and no measurements. A Cox proportional hazards model was used to determine the association between kidney function and mortality, controlling for demographics, Charlson morbidity scores, hypertension, and visits to general practitioners.

## Results

Of the 22,051 new users of metformin, 1,766 (8.0%) did not have a creatinine measurement before or after metformin initiation. Baseline characteristics were similar across groups stratified by frequency of creatinine measurements. A larger proportion of patients who had no creatinine measurements were rural-residing (31.3%) compared to those with pre (17.5%), post (21.1%) and both (15.8%) measurements. Of the subset with a measured eGFR, 25% of incident metformin users had eGFR <60ml/min. There was a small significant increase in the risk of mortality in incident metformin users with CKD stage 3b only (HR: 1.15 ; 95% CI: 1.04-1.28) compared to people with an eGFR of ≥60 mL/min/1.73m2.

## Conclusions

Metformin was commonly prescribed to patients with decreased kidney function, and did not appear to be associated with an increased risk of death.

